# EEG difference in the Higuchi fractal dimension of wakefulness and sleep from birth to adolescence

**DOI:** 10.1371/journal.pone.0333903

**Published:** 2025-10-13

**Authors:** Francesco Colussi, Jacopo Favaro, Claudio Ancona, Edoardo Passarotto, Maria Federica Pelizza, Eleonora Lorenzon, Simone Ruzzante, Stefano Masiero, Giorgio Perilongo, Giovanni Sparacino, Irene Toldo, Stefano Sartori, Maria Rubega

**Affiliations:** 1 Department of Information Engineering, University of Padova, Padova, Italy; 2 Women’s and Children’s Health Department, University of Padova, Padova, Italy; 3 Department of Neuroscience, University of Padova, Padova, Italy; Belgrade University Faculty of Medicine, SERBIA

## Abstract

Brain maturation from birth to adolescence involves profound transformations in neural dynamics, which can be studied in a minimally invasive manner using quantitative EEG. Most of the results published in the literature are based on spectral analysis approaches, which are extremely effective in detecting and assessing EEG rhythms. However, some aspects of EEG dynamics can only be investigated using nonlinear approaches, the use of which is still relatively unexplored in the pediatric population. The aim of the present paper is to assess the EEG differentiation of wakefulness from deep sleep (quiet sleep in neonates, stage N3 in older children) and its maturation across a wide developmental window (0–17 years) using the fractal dimension. Specifically, Higuchi fractal dimension (HFD) algorithm is used to analyse both wakefulness and sleep EEG recordings collected from 63 infants (aged 0-1 year) and 160 children (aged 2-17 years). To ensure methodological consistency, a data-driven criterion for the selection of HFD user parameters is implemented to enhance reproducibility. Our results show that HFD during wakefulness increases during the first year of life, followed by a stabilization or slight decrease in later years. In contrast, HFD during sleep exhibits a more stable profile, with only a mild increase over development. These findings are consistent with known neurodevelopmental processes—including synaptogenesis, pruning, and white matter maturation—and support the interpretation of HFD as a sensitive marker of large-scale integrative brain dynamics. These physiological trajectories of HFD both in wakefulness and sleep could be used as reference for future clinical applications in pediatric neurology and developmental monitoring.

## Introduction

**Fig 1:**
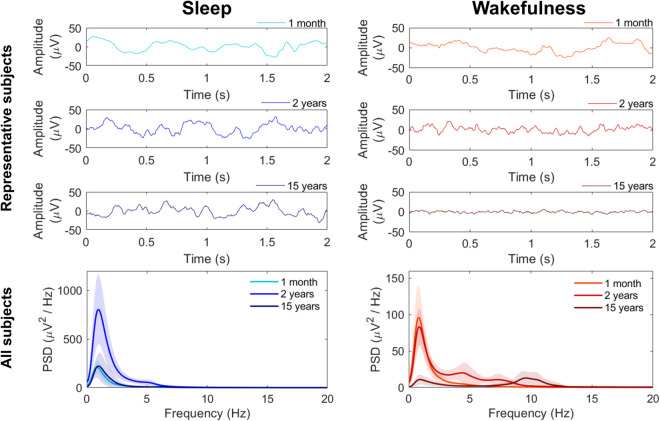
EEG during sleep vs EEG during wakefulness across development. EEG signals recorded at electrode C3 during sleep (left column) and wakefulness (right column) in three representative subjects of different ages: 1 month (first row), 2 years (second row), and 15 years (third row). The C3 electrode was chosen as a representative example because it is located in a central region of the scalp that is generally less affected by artifacts. We report signals of 2-s duration because Higuchi Fractal Dimension was calculated in 2-s EEG epochs. The last row shows the average power spectral density (thick line) and its standard deviation (shadow) for all subjects aged 1 month, 2 years and 15 years.

Throughout infancy, childhood, and adolescence, the brain undergoes significant functional and structural maturation [1–4]. One of the most noticeable and quantifiable aspects of this development is the evolution of EEG activity both in sleep and wakefulness. In particular, during wakefulness, the distribution of EEG power across frequency bands evolves in parallel with the maturation of sensory, motor, and cognitive systems, becoming increasingly organized and defined with age [5–9]. To get a grasp of EEG changes with age during wakefulness, three representative recordings are displayed in Fig 1, right column.

During sleep, the cortical electrical activity is dominated by slow wave activity whose characteristics vary across development, in step with cortical maturation. This slow wave activity reflects synaptic density, synaptic strength and synaptic efficacy, and are considered reliable markers for plastic changes during childhood and adolescence [10–14]. To qualitatively show the small changes in frequency during sleep as age increases, three representative recordings are displayed in Fig 1, left column (same subjects as in right column).

Traditionally, maturational aspects of EEG have been quantitatively studied using spectral analysis techniques, e.g., through discrete Fourier transform or autoregressive models [15] (e.g., Fig 1, last row). However, given the composite nature of EEG, nonlinear methods should also be exploited to provide further insights and complementary understanding of brain activity and neurodevelopment [16–18]. Several nonlinear techniques, including fractal dimension, approximate entropy and the Hurst exponent, were applied in the analysis of adults sleep EEG, particularly in distinguishing between different NREM sleep stages [19]. Nonlinear indices were also combined with machine learning algorithms to achieve automated detection of sleep stages based on EEG data [20]. Despite these advances, the use of nonlinear approaches to study EEG in the pediatric population is still relatively unexplored. Nonlinear indices, respectively, fractal dimension and multiscale entropy, demonstrated to complement power spectral analysis in the context of studying sleep in neonates [21] and memory task in adolescents (8-15 y) and young adults (20-33 y) [22].

Among the various nonlinear signal analysis techniques, Fractal Dimension (FD) holds promise for studying pediatric EEG. A first potential advantage of FD is that it provides a global description of a signal’s complexity, which is valuable for understanding overall brain activity [23–25]. Then FD analysis allows the assessment of signal dynamics within the time domain, making it easier to correlate EEG variations with changes in complexity [23] and to capture signal-specific characteristics within short time windows [26]. FD is based on self-similarity [27] and this feature is especially effective at characterizing recurrent and regular patterns, highlighting EEG changes across different ages and brain states (such as sleep and wakefulness). Additionally, FD is independent of scale [25], this makes the index well-suited to capturing the rapid and significant changes that occur in the developing pediatric brain across different temporal and spatial scales. Finally, FD is also less sensitive to background noise and physiological artifacts—a common issue in pediatric EEG—and more robust to inter-individual variability [28].

Among the various methods for estimating FD from a time series, Higuchi Fractal Dimension (HFD) is highly regarded for its accuracy [29], being capable of providing accurate estimates, even for short segments [28,30].

Regarding pediatric populations, HFD showed promising results starting from neonatal studies where it demonstrates a significant correlation with gestational age and the capacity of discriminate wakefulness from quiet sleep in the first days of life [21,31]. When examining pediatric sleep EEG, there is evidence that HFD can effectively differentiate between sleep stages. HFD values were highest during wakefulness, followed by REM, N2 and N3 sleep stages, with a consistent decrease in complexity across these stages [32]. However, no prior studies have systematically investigated whether this differentiation evolves across the full pediatric age range. Despite HFD being effectively exploited across various age groups in both wakefulness and sleep [33], there is no agreed methodology to correct choice HFD tuning parameter (*k*_*lin*_). In the state-of-the-art literature, it has been proposed that the point at which the HFD reaches a plateau should be identified as the saturation point and that the corresponding *k* should be selected as the optimal value [34,35]. However, seeking a convergence of the computed HFD to a plateau is generally not a valid procedure, as not all data show that the HFD estimate reaches a plateau [36]. Accurately calculating fractal dimension can be a delicate process influenced by both the method used and the nature of the data [36]. For this reason, it is essential to use a data-driven approach to estimate the tuning parameter before computing the fractal dimension.

Beyond the methodological rationale, no unified biophysical model has yet explained the precise neurophysiological meaning of HFD [25]. Recent theoretical and empirical studies suggest that fractal measures may reflect the presence of scale-free, critical brain dynamics which are thought to emerge from self-organized criticality, a regime balancing order and disorder to optimize information flow and processing across spatial and temporal scales [25]. Based on this rationale, we hypothesize that the Higuchi Fractal Dimension (HFD), could reflect age-related maturation of large-scale brain network dynamics, with distinct profiles across vigilance states. Specifically, we hypothesize: (i) a progressive increase of HFD during wakefulness reflecting the development of integrative cortical dynamics; (ii) a relatively stable or less pronounced trajectory during NREM sleep, where large-scale brain activity is known to become more stereotyped and synchronized.

To test these hypotheses, the aim of the present paper is to map the developmental trajectories of HFD from birth to adolescence, during both wakefulness and sleep to provide a framework for future comparisons with pathological conditions. In particular, HFD is calculated in both sleep (as N3 sleep for subjects aged over three month and as quiet sleep for newborns) and wakefulness EEG signals in a large cohort of healthy subjects (223) aged between 0 and 17 years. Its evolution throughout infancy and adolescence is assessed by linear mixed effects regression models. The extensive age range permits a comprehensive examination of EEG maturation across pivotal developmental stages, from infancy to adolescence. To reduce the risk of introducing subjectivity in the results, a methodology has been proposed to select HFD user parameters in a reproducible manner.

## Materials and methods

### Dataset

This study included 63 infants (49% female; aged 0–1 years; dataset 1) and 160 children (41% female; aged 2–17 years; dataset 2) who underwent a 60-min EEG recording at the Pediatric Neurology and Neurophysiology Unit of the Department of Women’s and Children’s Health at the University Hospital of Padova. All participants were neurologically healthy and had typical psychomotor development. EEG recordings were performed after acquiring written informed consent from the participants’ parents. All data were fully anonymized before being shared for research purposes, in accordance with ethical and data protection guidelines. The data were accessed for research purposes between 25/02/2022 and 04/04/2024. The authors did not have access to any information that could identify individual participants at any stage of the research. The Ethics Committee for Clinical Trials of the University Hospital of Padova took note of this retrospective study (reference number 214n/AO/22).

Inclusion Criteria:

- Age between 0 and 17 years of age;
- Born at full term;
- EEG reported as normal;
- Typical neurological development, with no ongoing neurological issues and no neurological problems identified during follow-up at the same hospital;
- EEG recordings obtained from at least 10 electrodes placed according to the International 10-20 System;
- EEG recordings with at least 4 minutes of quiet wakefulness without artifacts and/or, if sleep was achieved, at least 4 minutes of NREM sleep (stage N3) for infants, children and adolescents and of quiet sleep for newborns.

Exclusion Criteria:

- Pathological EEG recordings in the previous 6 months;
- Diagnosis of neurodevelopmental disorders;
- On medication that could potentially modify the EEG signal (i.e., benzodiazepines, steroids).

### Data recording

EEG recordings were acquired using 10 electrodes (Fp1, Fp2, Fz, C3, C4, Cz, T3, T4, O1, O2) in Dataset 1, and 19 electrodes (Fp1, Fp2, F3, F4, C3, C4, P3, P4, O1, O2, F7, F8, T3, T4, T5, T6, Fz, Cz, Pz) in Dataset 2, with a Galileo MIZAR - Sirius amplifier (EBNeuro, Florence, Italy). Electrodes were placed according to the international 10–20 system, with the reference electrode positioned between F3 and F4, and impedance maintained below 5 kΩ. Signals were sampled at 256 Hz and filtered online between 0.1–70 Hz. All EEG recordings included synchronized video monitoring and additional polygraphic channels (e.g., deltoid EMG, ECG, and pneumogram in neonates) to support behavioral state classification, following published pediatric EEG protocols [37–39]. Video recordings were actively used to confirm eye opening/closure, spontaneous motor activity, and behavioral responsiveness, thereby ensuring accurate identification of wakefulness and allowing exclusion of ambiguous states.

Recordings were performed during routine clinical evaluations, typically in the morning or early afternoon. In infants under 1 year, sessions were scheduled after feeding to increase the likelihood of capturing quiet wakefulness, in line with the above-mentioned protocols [37–39]. During wakefulness, infants were held or placed supine in a dimly lit, quiet room with a parent present, and no active stimulation was provided. Recordings lasted at least 60 minutes to maximize the probability of capturing both wakefulness and sleep. In many cases, the session was extended until both a clearly defined phase of quiet wakefulness and a consolidated phase of NREM sleep were recorded.

No sleep deprivation was used, to avoid introducing confounding effects. In neonates, infants, and toddlers, sleep was facilitated by timing recordings after feeding. When necessary, sleep onset was promoted using fast-acting melatonin (1–3 mg orally), in accordance with standard pediatric clinical practice and with parental consent [37–39]. Environmental optimization was applied to promote sleep (dark room, minimal noise, and, when available, familiar relaxing music brought by the family). Infrared video-EEG allowed continuous monitoring in low-light conditions.

### Data pre-processing

Recorded data were imported and analyzed using custom MATLAB code (MATLAB R2023a, The MathWorks Inc., Natick, MA, USA).

First, for each participant, EEG data were high-pass filtered at 0.5 Hz with a 5th order Butterworth, then low-pass filtered at 60 Hz with a 3rd order Butterworth filter, avoiding phase distortion. A 50-Hz Butterworth 2nd order notch filter was applied to remove line noise.

According to current AASM guidelines (version 3.1; [40]), alternative references to mastoid reference such as Cz or average montage were used to identify graphoelements (e.g., spindles, K-complexes, slow waves). Epochs were selected by board-certified pediatric neurophysiologists using established behavioral and electrophysiological criteria. Sleep stages were classified according to AASM criteria adapted for pediatric populations [40]. In neonates, only quiet sleep (QS)—the developmental precursor of NREM sleep—was analyzed, based on EEG patterns (e.g., tracé alternant), respiratory activity, and behavioral state. Active sleep was excluded because: (i) it corresponds functionally to REM sleep, which is rarely observed in daytime EEG in older children, and (ii) its identification is unreliable without EOG/EMG. In infants <2 months of age, Stage N (undifferentiated NREM) was included, as graphoelements such as spindles and K-complexes are typically absent. In children older than 2 months, only Stage N3 epochs were selected for analysis. Scoring sleep stages in infants presents specific challenges. In the first months of life, N1 is difficult to identify due to the late appearance of vertex sharp waves (typically after 5–7 months), and hypnagogic hypersynchrony with high-amplitude slow waves is often observed instead. Moreover, in infants, the standard amplitude-based criteria for distinguishing N3 are less reliable, as high-amplitude slow waves may also appear in early N2. For these reasons, we analyzed quiet sleep in neonates and N3 in infants and children, selecting the most stable and recognizable NREM phases at each age (please, see also S1 Appendix).

Lastly, all EEG recordings were re-referenced to the average reference and segmented in 2-s nonoverlapping epochs (512 samples). Each epoch used for analysis was homogeneous in terms of vigilance state, avoiding contamination from transitional or mixed patterns.

The general characteristics of the dataset after preprocessing are summarized in Table 1.

**Table 1:** Preprocessed data used for further analysis. The table reports the number of subjects for each age category with at least 4-min artifact-free wakefulness and sleep EEG, and the range of 2-s artifact-free epochs for each vigilance stage. The columns represent (from left to right): the age interval; the total number of subjects ($N_{sbj}$); the number of subjects with at least 4-min artifact-free wakefulness EEG ($N_{sbj}$ **(W)**); the number of subjects with at least 4-min artifact-free sleep EEG ($N_{sbj}$ **(S)**); the number of 2-s artifact-free EEG wakefulness epochs per subject [min–max] **(W epochs)**; the number of 2-s artifact-free EEG sleep epochs per subject [min–max] **(S epochs)**.

Age interval	$N_{sbj}$	$N_{sbj}$ (W)	$N_{sbj}$ (S)	W epochs	S epochs
[0, 1] months	23	23	21	[120, 262]	[120, 626]
[2, 12] months	40	32	27	[120, 411]	[120, 578]
[2, 4] years	30	30	20	[120, 702]	[120, 679]
[5, 8] years	40	40	16	[120, 725]	[120, 387]
[9, 12] years	40	40	6	[120, 622]	[120, 329]
[13, 17] years	50	50	3	[120, 607]	[120, 241]

**Fig 2:**
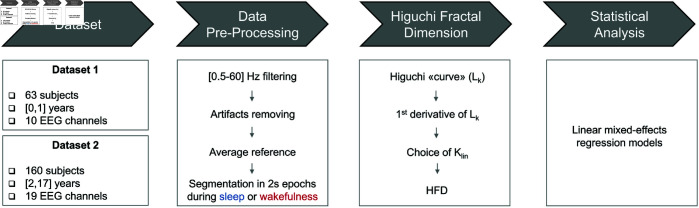
Graphical summary of the data processing pipeline.

An overview of the EEG processing workflow is shown in Fig 2.

### Higuchi fractal dimension

HFD is an indicator computed in the time domain. By definition, its value can range between 1 and 2, with higher values indicating a more irregular signal. For a comprehensive description of HFD computation we refer the reader to [41]. Briefly, the procedure is as follows.

For each sample *i* of the EEG epoch x=x(1),x(2),x(3),…,x(N) of length *N*, the absolute differences between values *x*(*i*) and *x*(*i*–*k*), i.e., the samples at distance *k*, are computed, considering $k=1,2,\ldots ,k_{lin}$.

Each absolute difference is multiplied by a normalization coefficient that takes into account the different numbers of samples available for each value of *k*. The computation of this coefficient is based on the starting point m=1,…,k and on the total number *N* of samples of an epoch. This normalized absolute differences are computed for each starting point *m* and all possible samples at distance *k* and this quantity is called *L*_*m*_(*k*)

$$L_{m}(k)=\frac{1}{K}\cdot \left[\sum_{i=1}^{q}\left|x(m+i\cdot k)-x(m+(i-1)\cdot k\right|\right]\cdot \frac{N-1}{q\cdot k}$$ (1)

where q=int[(N−m)/k].

For each $k=1,2,\ldots ,k_{lin}$, L(k) is computed by summing the obtained *L*_*m*_ values and dividing by *k*. Then, the *log*[*L*(*k*)] vs *log*(*k*) curve, called *l*_*k*_, is derived. By definition, if *L*(*k*) is proportional to *k*^−*D*^ (i.e., log(k) and log[L(k)] have a linear relationship) for $k=1,2,\ldots ,k_{\text{lin}}$, then the curve is fractal with dimension *D*. Consequently, $k_{\text{lin}}$ is the maximum *k* for which *L*(*k*) is proportional to *k*^−*D*^ and *D* is estimated by ordinary least squares as the linear coefficient of the regression line (slope) of the logL(k) curve for $k=1,2,\ldots ,k_{\text{lin}}$. The lack of a standardized method in selecting the value of the parameter *k*_*lin*_ has resulted in great variability and limited comparability in published studies [42]. For this reason, there is a growing need for a rationalized and reliable criterion to choose the parameter in order to align methodological pipelines [36]. We selected the value of *k*_*lin*_ evaluating the properties of the curve *l*_*k*_ as a function of the scale *k*. Standardizing the selection of the parameter *k*_*lin*_ in the calculation of HFD permits computation of comparable HFD values across different subjects and conditions.

HFD is based on the assumption that, on a logarithmic scale, the relationship between *log*[*L*(*k*)] and *log*(*k*) is linear over a range of k values. This linearity reflects the self-similarity of the time series, a key characteristic of fractal structures [27]. If *k*_*lin*_ is chosen correctly, the linear fit on the *log*[*L*(*k*)]/*log*(*k*) curve will be accurate, and the slope of this line will provide a good estimate of the fractal dimension. Higher values of *k*_*lin*_ may extend the linear fit beyond the region where the logarithmic relationship is linear. In such cases, the calculated slope will no longer solely reflect the self-similarity of the series, but will be influenced by other characteristics of the time series such as periodicity or other statistical properties, distorting the fractal dimension estimate. Therefore, it is crucial to select a *k*_*lin*_ value that allows for calculating the linear fit of the *log*[*L*(*k*)]/*log*(*k*) curve on the initial *k* points, where the curve’s behavior is still linear. If this linear relationship does not hold, the signal does not have fractal properties. We systematically evaluated how the linearity of this relationship varies with increasing k across our entire data set to ensure that linearity was maintained at the k interval chosen for analysis.

**Fig 3:**
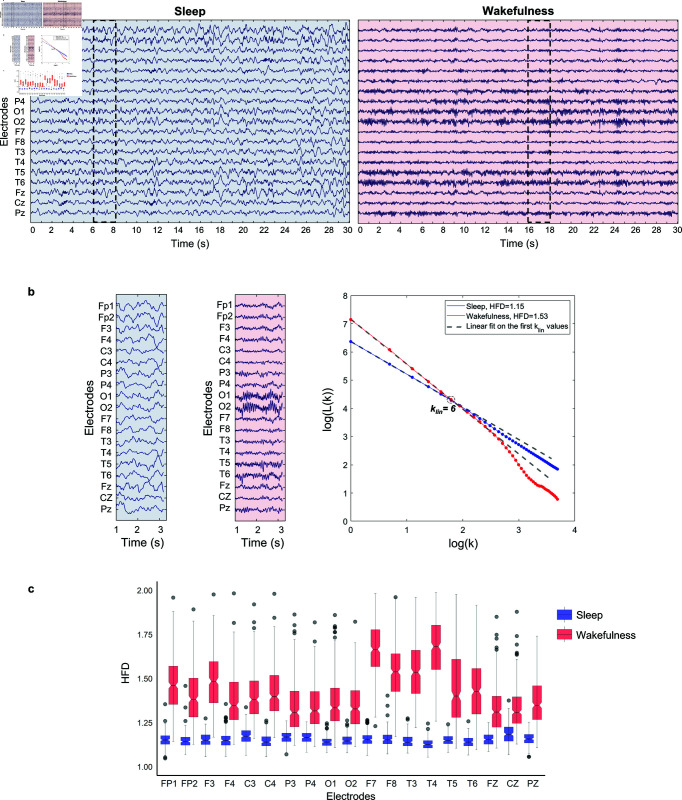
Processing of wakefulness and sleep EEG data to calculate the HFD in a representative subject. (a) 30-s EEG recording for sleep (in blue) and wakefulness (in red) in a representative 11-year-old subject. The dashed black boxes mark 2-s epoch free of artifacts. (b) 2-s epochs free of artifacts for sleep (in blue) and wakefulness (in red), with the corresponding *l*_*k*_ curves. The blue and red dots represent the values of the *l*_*k*_ curve as *k* increases. The dashed grey line represents the linear fit across the initial *k*_*lin*_ points, with its slope indicating the HFD. (c) Boxplots of the distribution of all HFD values for each electrode obtained for each sleep (in blue) and wakefulness (in red) epoch.

In the present study, HFD was calculated for each 2-s epoch (512 samples) and the HFD values obtained from each epoch were averaged at the electrode-level to obtain a single value that describes the signal complexity for each electrode (Fig 3).

To evaluate up to which value of *k*_*lin*_ the behavior of the *l*_*k*_ curve is linear, we calculated its first and second derivative (the first derivative of a straight line should be constant and the second derivative should be zero). *k*_*lin*_ corresponds to the value where the first derivative stops to be a constant and the second derivative to be zero. Specifically, for each 2-s epoch, we computed the derivatives $\left(\frac{d\log(L(k))}{d\log(k)},\frac{d^{2}\log(L(k))}{d\log(k){\;\;}^{2}}\right)$ with *k* from 1 to 40. The average derivative across all epochs for each subject provided the value of the derivative of the *l*_*k*_ curve for wakefulness and sleep EEG traces. We, then, averaged the derivative values of the *l*_*k*_ curves by age groups (0-1 years, 2-4 years, 5-8 years, 9-12 years, 13-17 years). We selected *k*_*lin*_ as the value where the first derivative was changing no more than 5% compared to the moving average of values with k from 1 to *k*_*lin*_-1 also visually checking that the second derivative was approximately zero. We took the average *k*_*lin*_ among the age groups. We did not evaluate higher k values than 40, as the linearity was already lost for k values below 40 (*k* > 11).

### Linear mixed effects regression models

Linear mixed-effects regression models were used to assess the effect of age and state (i.e., wakefulness vs sleep) on HFD. Due to the different measures of age and developmental period considered, the analyses were performed separately on the two datasets (i.e., subjects below and above the age of 52 weeks). HFD values were regressed on age and state, which represented the fixed-effect part of the model. Random intercepts per subject (subj) and EEG channel (ch) were modelled and the effect of age was allowed to vary across ch levels. Therefore, HFD was modelled as the following distribution:


$$\text{HFD}∼N(\beta_{0}\text{intercept}+\beta_{1}\text{age}+\beta_{2}\text{state}+\beta_{3}\text{age:state}+u_{\text{1,subj}}+u_{\text{2,ch}}+u_{\text{3,ch}}\text{age},\sigma^{2})$$

Random effects were modelled as zero-mean distributions:


$$u_{i}∼N(0,\tau_{i})$$

Age values were normalized to the 0-1 range and state was coded as 0 = awake and 1 = asleep. Model summaries are reported together with marginal and conditional R^2^ values, estimating the amount of variance explained by the model at a population (i.e., across subjects and EEG channels) as well as at the individual (i.e., for each subject and EEG channel) levels, respectively. The analyses were performed in RStudio using the lme4 R package [43].

## Results

We selected a *k*_*lin*_ value of 6 as an adequate value for effectively calculating HFD for a population aged 0 to 17 years with a sampling rate of 256 Hz (see S2 Appendix for further details).

**Fig 4:**
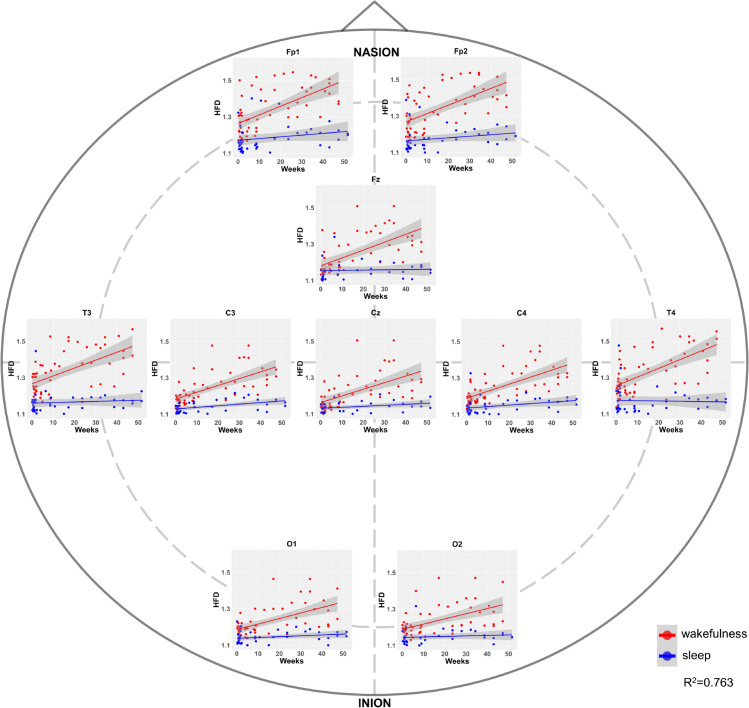
Progressive differentiation of wakefulness from sleep in terms of EEG fractal dimension from 0 to 1 year of age. The figure shows average HFD values for each subject in Dataset 1 at the electrode level. Red dots represent the average HFD values for each subject during wakefulness, while blue dots represent the values during sleep. Both red and blue lines indicate the respective regression lines in wakefulness and sleep. The X-axis displays the subjects’ age in weeks. The figures are arranged to show the positions of the electrodes on the scalp where the data were acquired.

**Fig 5:**
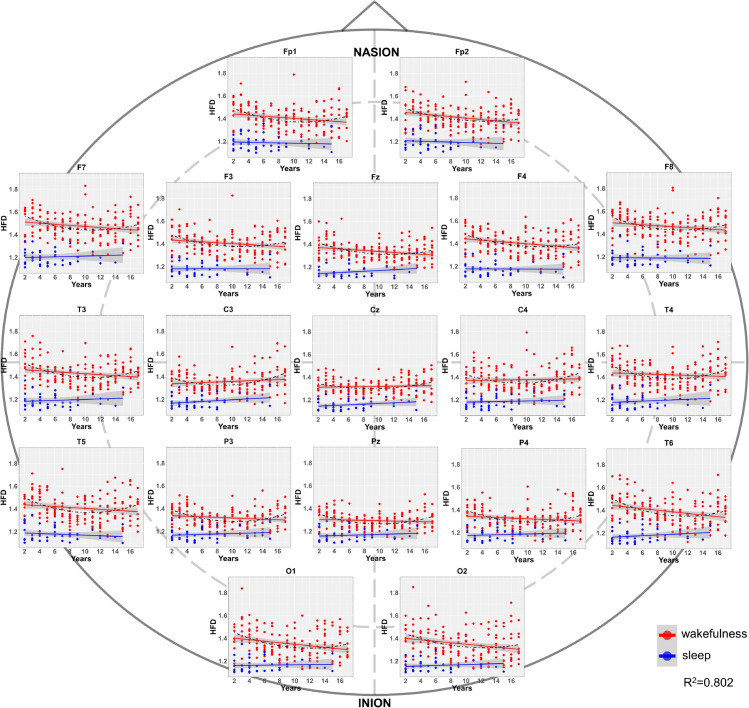
Progressive differentiation of wakefulness from sleep in terms of EEG fractal dimension from 2 to 17 years of age. The figure shows average HFD values for each subject in Dataset 2 at the electrode level, consistent with Fig 4. The X-axis displays the subjects’ age in years. The quadratic fit on the HFD values during wakefulness is also shown with a dark-gray dashed line for each electrode.

Figs 4 and 5 represent the average HFD values per electrode for all subjects in Dataset 1 and Dataset 2. In all the 10 electrodes of Dataset 1, a progressive maturation of EEG signal irregularity during wakefulness is observed, with HFD values gradually increasing with age. The sleep EEG also shows an increase in irregularity, but this growth is much smaller compared to wakefulness. In newborns (0-1 month of age), the HFD values for sleep and wakefulness are very close, and in some electrodes, they even overlap. The significant maturation of EEG signal irregularity after the first month of life results in a clear differentiation of wakefulness and sleep HFD values as age increases, even within the first year of life. In the subjects from Dataset 1, the channels with the highest fractal dimension values in wakefulness appear to be the temporal ones (T3 and T4). In Dataset 2, the HFD values for 2-year-old subjects start at around a 1.4 which is approximately the value reached by the older subjects in Dataset 1. Then, as age increases, a slight decreasing trend in HFD is observed across almost all 19 electrodes until around the age of 8 years, after which the HFD values begin to rise again, reaching peak complexity values of approximately 1.8 around 16-17 years of age. Similar to Dataset 1, the HFD values during sleep show a very slight increase, but overall, the sleep HFD values remain quite consistent from 5 months to 17 years. In Dataset 2, the two distributions of values are substantially stable with age, allowing a discrimination between wakefulness and sleep cortical electrical activity. In this dataset as well, the channels showing the highest fractal dimension values during wakefulness were located over the temporal regions (T3 and T4), as well as over the lateral frontal areas (F7 and F8), approximately corresponding to the dorsolateral prefrontal cortex, which was not included in the EEG montage used for infants.

The results of the mixed effect regression models confirm quantitatively what was qualitatively observed in Figs 4 and 5 and are summarized in Table 2. Table 2 reports that the state (i.e., wakefulness and sleep) had a significant and negative main effect on HFD in both subgroups. Age was significantly associated with an increase in HFD during wakefulness in subjects aged 0-1 year, while a less pronounced decrease in HFD was observed during wakefulness in subjects aged 2-17 years. Age:state interactions were significant for both subgroups, but in opposite directions. This indicates that, during sleep, both the 0-1 year and 2-17 years age groups show a slight increase in HFD with age; however, this increase is a bit more pronounced in the 0-1 year group. The models explained substantial amounts of variance in HFD values, with conditional ${\text{R}}^{2}=0.762$ and marginal ${\text{R}}^{2}=0.430$ for 0-1 year group and conditional ${\text{R}}^{2}=0.802$ and marginal ${\text{R}}^{2}=0.382$ for 2-17 years group. These R^2^ values indicate the robustness of the model, possibly strengthening the credibility of our results.

**Table 2:** The effect of age, state (i.e., wakefulness and sleep), and age:state interaction on HFD values (N = 223). The table reports fixed effects estimates from the linear mixed effects models used in the analyses.

Variable	Age < 52 weeks (N = 63)				Age > 52 weeks (N = 160)			
	β	SE	t-value	p-value	*β*	SE	t-value	p-value
intercept	1.216	.014	82.186	<.001	1.409	.016	90.383	<.001
age	.187	0.023	8.091	<.001	–0.055	.021	–2.55	0.0014
state	–0.082	0.005	–17.04	<.001	–.271	0.04	–60.817	<.001
age:state	–.107	.14	–7.789	<.001	0.098	0.015	6.481	<.001

Given the nonlinear trend evidenced in Fig 5 for the state of wakefulness in the 2-17 years age group, a post-hoc quadratic mixed effects regression model (quadratic_fit) was fitted on this subset of the data and compared to a linear one (linear_fit). As shown in Table 3, the quadratic model resulted superior in terms of AIC, logLik, and chi-square values (p < .01). However, BIC values favoured the simpler linear model, likely due to the lower number of parameters fitted.

**Table 3:** Comparison between linear and quadratic regression models. Npar = number of model parameters, AIC = Akaike information criterion, BIC = Bayesian information criterion, logLik = log-likelihood, chi_sq = chi-squared, **Δ** = difference between regression models.

model	npar	AIC	BIC	logLik	chi_sq	Δ chi_sq	Δ npar	p (Δ chi_sq)
linear_fit	7	–8044.5	–8002.3	4029.2	–8058.5			
quadratic_fit	11	–8052.5	–7986.2	4037.2	–8074.5	15.986	4	0.003

To further explore potential topographical differences in HFD, we conducted an additional linear mixed-effects analysis focusing on a region of interest (ROI) encompassing the frontal (F7, F8) and temporal (T3, T4) electrodes—channels previously noted to display higher HFD values during wakefulness. In infants younger than 52 weeks, only T3 and T4 were included in the ROI due to limitations in the EEG montage. The model revealed a significant age × ROI interaction in younger subjects, indicating a faster increase in HFD over temporal areas relative to other scalp regions. In older children, we observed a significant main effect of ROI, with consistently higher HFD values in frontal and temporal areas compared to the rest of the scalp. Full model specifications and statistical outputs are reported in S3 Appendix.

## Discussion

Our study provides relevant findings from both a methodological and clinical perspective.

From a methodological perspective, we introduce a repeatable and standardized pipeline for calculating the Higuchi Fractal Dimension in pediatric EEG signals. The selection of the key parameter *k*_*lin*_ was mathematically justified through an analysis of the log-linear relationship in the *l*_*k*_ curves. This standardization is essential for ensuring that HFD values are comparable across different subjects, conditions and studies, thereby reducing variability and increasing the reliability of fractal analysis in pediatric EEG research.

From a clinical perspective, our study is, to our knowledge, the first to utilize fractal dimension analysis to examine pediatric EEG signals recorded during both wakefulness and sleep across a broad age range from 0 to 17 years. Most previous studies were limited to narrower age windows or focused on the discrimination between vigilance states [42]. We traced the developmental trajectory of HFD during both sleep and wakefulness by using a resolution of 1 month for the first year of life and 1 year for ages 2 to 17, providing a dynamic and integrated view of EEG maturation that is currently lacking in the literature.

During the first year of life, HFD shows a marked increase during wakefulness, which then stabilizes. In contrast a slight increase is observed during sleep. This developmental trajectory highlights a progressive differentiation between wakefulness and sleep during the first year of life. On the other hand, in older children, HFD demonstrates a more stable pattern, with consistent HFD values across both states and a clear distinction between wakefulness and sleep. Notably, we observed median HFD values that are consistent across the two subgroups. Specifically, the HFD values for both wakefulness and sleep increase during the first year of life, reaching values comparable to those found in 2-year-old. From a translational perspective, mapping physiological developmental trajectories of HFD during both wakefulness and sleep may provide useful reference framework for future studies aimed at detecting potential different HFD trajectories in pathological conditions.

Moving beyond the establishment of descriptive reference values, our findings may also offer insights into the neurophysiological processes underlying brain development. While the precise physiological meaning of HFD remains incompletely understood and no unified biophysical model currently explains its developmental trajectory [25], several key processes underpinning early neurodevelopment are worth considering. In particular, the dynamic interplay between synaptogenesis, synaptic pruning, and white matter maturation has been widely implicated in shaping both structural and functional brain connectivity [1,4,13]. Previous studies have hypothesized that these biological processes contribute to a progressive increase in the efficiency of neural networks, ultimately reflected in the complexity of EEG activity [44–46]. Interpreting the observed wakefulness-related trajectory of HFD in this framework, the marked increase during the first two years of life likely may correspond to the phase of intense synaptogenesis, characterized by an overproduction of synaptic connections. Indeed, in children over the age of two, the regression line seems to suggest a slight decrease of HFD from childhood to adolescence. However, this decrease could be the result of a U-shaped trend in HFD during development which can be interpreted within the context of two fundamental neurobiological processes: from ages 2 to 8, gray matter development is dominated by synaptic pruning during which redundant connections are eliminated, potentially resulting in a temporary reduction in signal complexity [1,4,13,45–48]. As children approach 8-9 years of age, white matter maturation becomes more prominent than grey matter maturation, continuing into adulthood [49–51]. This process enhances the efficiency of neural connectivity, leading to a subsequent increase in EEG complexity [44,52]. Thus, the U-shaped trend may reflect the dynamic balance between synaptogenesis, pruning, and white matter maturation during development. In contrast, the developmental trajectory of HFD during sleep appears more stable. Given that HFD is sensitive to the self-similarity of the EEG signal, the slight increase observed during NREM sleep in infants may reflect the emergence of sleep transients such as vertex waves, spindles, and K-complexes. In older children, it may correspond to the gradual maturation and stabilization of macro-and microstructural features of physiological sleep [5,6,10,53]. Both of these processes could contribute to greater irregularity in sleep EEG signals across development.

This hypothesis needs to be corroborated by expanding the dataset since the number of EEG recordings containing at least 4 minutes of N3 NREM sleep in participants over eight years old is limited (9 subjects). This reflects a physiological and logistical limitation of routine clinical EEG recordings in older children and adolescents, where consolidated sleep—particularly stages N2 and N3—is less likely to occur during short daytime or nap EEG sessions.

The age-dependent changes we observed in HFD during wakefulness may mirror the progressive transition from local, modular dynamics—typical of early infancy—to more integrated and distributed network activity in later childhood. This aligns with neurodevelopmental models suggesting that early brain function is dominated by relatively autonomous sensorimotor subsystems, which gradually evolve into a globally integrated and self-referential system [54–56]. An intriguing observation in this context is the higher HFD values recorded over fronto-temporal electrodes in both datasets. If replicated in future studies, this could suggest that temporal regions represent a “hot zone” of complexity across development. However, this interpretation must be treated with caution, as it remains unclear whether the observed effect reflects genuine neurophysiological activity or is partially influenced by residual artifacts. Younger children and infants are generally more prone to movement during EEG acquisition, which may lead to subtle muscular artifacts—particularly in temporal regions where cranial muscle activity is more prominent. Although all epochs with visible artifacts were carefully excluded during preprocessing, undetected or subthreshold noise could still have influenced complexity estimates. The observed increase in HFD during wakefulness may thus reflect the emergence of large-scale network coordination, supporting more integration of information and more flexible brain operations. Deviations from the normative trajectories might signal delays or disruptions in the maturation of large-scale neural connectivity, offering a promising tool for the early identification of atypical neurodevelopmental patterns (coherently with neuroimaging research, see for a review [57]).

Overall, our findings partially confirmed the initial hypotheses. In fact, while our hypothesis of a progressive increase in wakefulness HFD was confirmed in early infancy, it was not fully sustained across the entire pediatric age range. On the other hand, the developmental trajectory of HFD during NREM sleep was relatively stable, with only a slight age-related increase during the first year of life, supporting our expectation of reduced complexity dynamics during sleep compared to wakefulness.

This study has some limitations that must be acknowledged. First, the non-homogeneous distribution of EEG epochs across different ages and states poses a challenge. Second, the EEG data were obtained from different subjects at various ages, rather than tracking the same individuals longitudinally. This cross-sectional design should be taken into consideration when interpreting the observed trends in HFD, as it may introduce interindividual variability in developmental trajectories. Finally, the use of low-density EEG limits the study’s ability to thoroughly analyze topographical brain activity. Higher-density EEG would allow for more detailed spatial analyses and a better understanding of the underlying neural mechanisms contributing to the observed changes in HFD.

## Conclusion

This study provides, a comprehensive characterization of EEG Higuchi Fractal Dimension across wakefulness and NREM sleep from birth to adolescence and a methodology for calculating the HFD of EEG signals in pediatric subjects of different ages. An objective criterion was proposed to choice HFD user parameters in order to ensure repeatability and comparability. Results obtained in 223 subjects offered valuable insights into the maturation of brain from the neonatal period to adolescence: on the one hand, HFD increases with age during wakefulness, with a significant rise in the first year of life, followed by a stabilization thereafter; on the other hand, sleep EEG exhibits a much more stable pattern, with a slight increase from the first few months of life onward, remaining relatively constant through adolescence.

A major clinical value of our findings lies in the definition of normative developmental trajectories of EEG fractal dimension in healthy children. These trajectories can serve as benchmarks for detecting deviations in pathological conditions such as neonatal encephalopathy, stroke, neurodevelopmental disorders, disorders of consciousness, and broader pediatric neurological and psychiatric conditions. The observed patterns suggest that HFD captures meaningful age-dependent changes in brain dynamics, likely reflecting the dynamic balance between synaptogenesis, pruning, and white matter maturation but also the evolution of integrative neural processes both in wakefulness and in sleep. Future research using longitudinal designs, higher-density EEG recordings, and the computation of additional EEG features alongside HFD should be extremely useful in corroborating and expanding these findings. This research could have promising implications for basic neuroscience and clinical assessment in pediatric populations.

## Supporting information

S1 File Dataset1. The dataset1.csv is provided as supporting information and contains the average HFD values computed for each of the 10 electrodes and each subject during both wakefulness and sleep. Where no values were available, the dataset includes NaN entries. In addition to the HFD values for each electrode, the dataset includes the subject identifier, sleep/wakefulness state, and age expressed in weeks of life. These data were used to generate Figs 4 and 5 and are the inputs for the statistical analysis.
(ZIP)

S2 File Dataset2. The dataset2.csv is provided as supporting information and contains the average HFD values computed for each of the 19 electrodes and each subject during both wakefulness and sleep. In addition to the HFD values for each electrode, the dataset includes the subject identifier, sleep/wakefulness state, and age expressed in years of life. These data were used to generate Figs 4 and 5 and are the inputs for the statistical analysis.
(ZIP)

S3 File MATLAB code to generate the figure in S2 Appendix. (M)

S1 Appendix Sleep stage comparability. (PDF)

S2 Appendix *k*_*lin*_ parameter. (PDF)

S3 Appendix ROI-based mixed model. (PDF)
