# Supplementary material for: EEG difference in the Higuchi fractal dimension of wakefulness and sleep from birth to adolescence
Source: PLoS One. 2025 Oct 13;20(10):e0333903. doi: 10.1371/journal.pone.0333903 (PMC12517535; doi:10.1371/journal.pone.0333903)
Supplement: S1 Appendix — (PDF) [file pone.0333903.s004.pdf]

S1 Appendix: Sleep stage comparability.

To support the comparability of sleep stages across age groups, we report in Figure 7 the distribution of HFD values during sleep across developmental stages: quiet sleep in neonates, combined N2+N3 sleep in infants and N1, N2, N3 stages in children aged 2–17 years. As shown in the figure, HFD values in quiet sleep and in infant N2+N3 sleep closely overlap with those recorded during N3 in older children—while they differ from those observed in N1 and N2. This supports the comparability of our sleep stage selection across age groups.

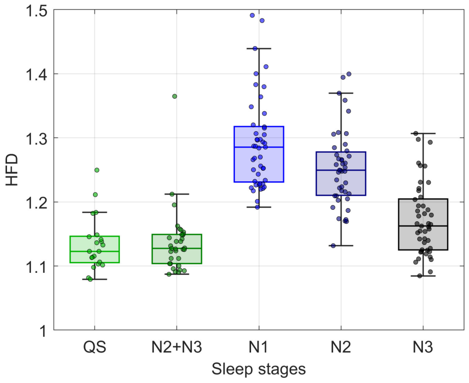

Figure 7: *Distribution of HFD values across different sleep stages and developmental groups.* Boxplots represent HFD values during quiet sleep (QS) in neonates (light green), combined N2+N3 sleep in infants (dark green), and N1, N2, and N3 sleep stages in children aged 2–17 years (in blue and grey). HFD values in QS and infant N2+N3 are comparable to those observed in N3 of older children, and are markedly lower than values observed in N1 and N2.

In addition, we repeated the main analyses in Dataset 2 (ages 2–17 years, N=160) using combined N2+N3 sleep epochs instead of N3 alone. As illustrated in Figure 8 and Table 4, the results were consistent with those reported in the main text, confirming the robustness of our findings.

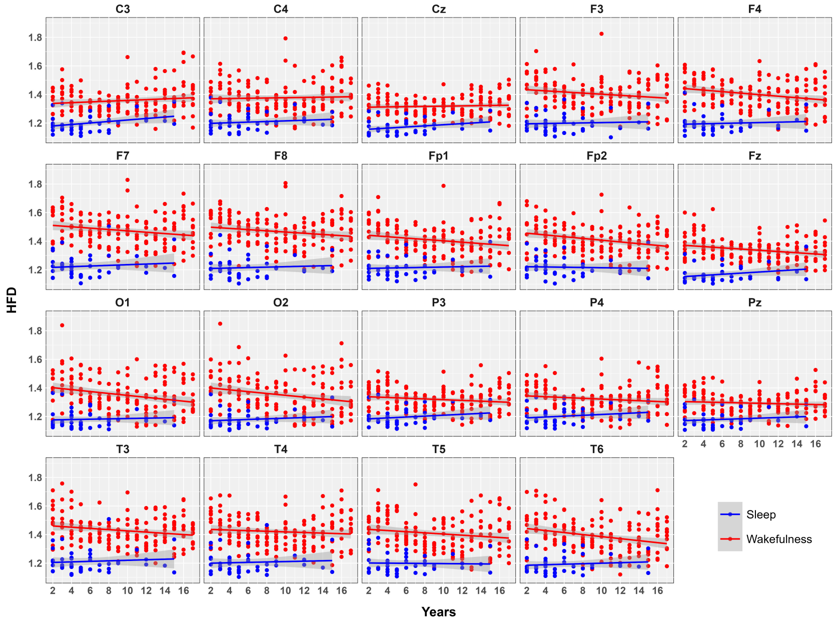

Figure 8: *Differentiation of wakefulness from sleep in terms of EEG fractal dimension from 2 to 17 years of age.* The figure (similarly to Fig 5 in the main text) shows average HFD values in wakefulness and NREM sleep (N2+N3) for each subject in Dataset 2 at the electrode level. Red dots represent the average HFD values for each subject during wakefulness, while light blue dots represent the values during sleep. Both red and light blue lines indicate the respective regression lines in wakefulness and sleep. The X-axis displays the subjects' age in years.

Table 4: *The effect of age, state, and age:state interaction on HFD values in subjects with age > 52 weeks (N=160) considering N2+N3 sleep.* The table reports fixed effects estimates from the linear mixed effects models used in the analyses.

Variable	β	SE	t-value	p-value
intercept	1.406	0.018	77.2	<.001
age	-0.052	0.022	-2.32	0.021
state	-0.258	0.011	-23.34	<.001
age:state	-0.259	0.013	-19.32	<.001
