# Supplementary material for: EEG difference in the Higuchi fractal dimension of wakefulness and sleep from birth to adolescence
Source: PLoS One. 2025 Oct 13;20(10):e0333903. doi: 10.1371/journal.pone.0333903 (PMC12517535; doi:10.1371/journal.pone.0333903)
Supplement: S2 Appendix — (PDF) [file pone.0333903.s005.pdf]

S2 Appendix: k_{lin} parameter.

The relationship between $\log[L(k)]$ and $\log(k)$ show a loss of linearity at progressively lower k values with increasing age, with a maximum k_{lin} value of approximately 8 (Figure 6, panels (b) and (d)). The sleep EEG traces indicate that the l_k curve maintains linearity longer compared to wakefulness traces. However, for subjects aged between 13 and 17 years linearity is lost around $k = 9$ (Figure 6, panels (a) and (c)). Based on these considerations, we selected $k_{lin} = 6$.

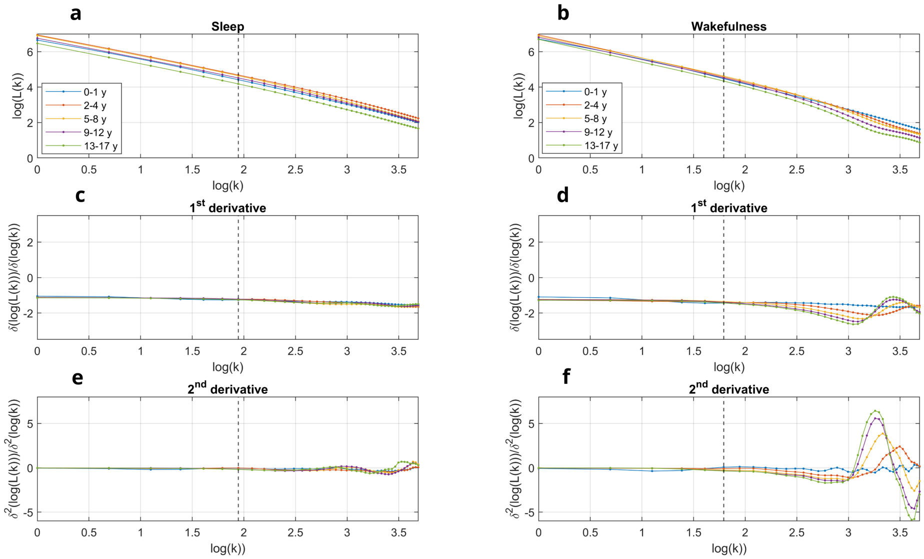

Figure 6: *Changes of l_k curve and of its first and second derivative across development.* (a) and (b) show the averaged values of the l_k curves by age groups (0–1 years, 2–4 years, 5–8 years, 9–12 years, 13–17 years) for sleep (a) and wakefulness (b). (c) and (d) show the average values of the first derivative of the l_k curves for the same age groups as in (a) and (b), sleep l_k derivatives in (c) and wakefulness in (d). (e) and (f) illustrate the second derivatives of the l_k curves during sleep and wakefulness, respectively. The bold points represent the values of $k = (1, 2, \ldots, 40)$. The dashed vertical black line shows the value of $k_{lin} = 6$, indicating that for each age group, the trend is linear at this value. The legend displays the color coding for the age groups.
