# Supplementary material for: EEG difference in the Higuchi fractal dimension of wakefulness and sleep from birth to adolescence
Source: PLoS One. 2025 Oct 13;20(10):e0333903. doi: 10.1371/journal.pone.0333903 (PMC12517535; doi:10.1371/journal.pone.0333903)
Supplement: S3 Appendix — (PDF) [file pone.0333903.s006.pdf]

S3 Appendix: ROI-based mixed model.

To statistically assess spatial differences in HFD across development, we defined a binary region-of-interest (ROI) variable that included frontal (F7, F8) and temporal (T3, T4) electrodes, coded as 1; all other electrodes were coded as 0. In infants (<52 weeks), only T3 and T4 were available in the 10-electrode montage and included in the ROI. We fitted an linear mixed effects model regressing HFD values on age, ROI, and their interaction (age × ROI), including random intercepts for subject and channel, and allowing the effect of age to vary by channel (Table. 5). The model showed a significant age × ROI interaction in infants, with a steeper increase in HFD over temporal regions. In children older than 52 weeks, a significant main effect of ROI was found, indicating persistently higher HFD in frontal and temporal channels. These results support a spatially differentiated maturation of brain signal.

Table 5: *Linear mixed effects model estimates for HFD as a function of age, binary ROI and their interaction, shown separately for infants (<52 weeks, N = 63) and older children (>52 weeks, N = 160).*

	Age < 52 weeks (N = 63)				Age > 52 weeks (N = 160)			
Variable	β	SE	t-value	p-value	β	SE	t-value	p-value
intercept	1.211	.018	66.937	<.001	1.388	.018	76.120	<.001
age	.185	0.034	5.453	<.001	-0.051	.0224	-2.08	0.039
ROI	0.024	0.028	.848	.420	0.087	0.027	3.142	<.001
age:ROI	0.088	0.030	2.872	0.021	-0.006	0.023	-0.293	0.773
